# Oxidative Stress and Annexin A2 Differential Expression in Free Fatty Acids-Induced Non-Alcoholic Fatty Liver Disease in HepG2 Cells

**DOI:** 10.3390/ijms25179591

**Published:** 2024-09-04

**Authors:** Vinícius Marques Arruda, Gabriela Tolentino Azevedo, Maria Júlia Maia Gonçalves Granato, André Carlos Pereira Matos, Thaise Gonçalves Araújo, Joyce Ferreira da Costa Guerra

**Affiliations:** 1 Laboratory of Metabolic Biochemistry and Redox Processes, Institute of Biotechnology, Federal University of Uberlandia, Patos de Minas 38700-002, Brazil; vinicius.arruda@ufu.br (V.M.A.); gabriela.azevedo@ufu.br (G.T.A.); maria.granato@ufu.br (M.J.M.G.G.); 2 Laboratory of Genetics and Biotechnology, Institute of Biotechnology, Federal University of Uberlandia, Patos de Minas 38700-002, Brazil; andre.carlos@ufu.br (A.C.P.M.); tgaraujo@ufu.br (T.G.A.)

**Keywords:** hepatic steatosis, HepG2 cells, inflammation, lipotoxicity, Keratin 17

## Abstract

Non-alcoholic fatty liver disease (NAFLD) is a rising global burden, affecting one in four adults. Despite the increasing prevalence of NAFLD, the exact cellular and molecular mechanisms remain unclear, and effective therapeutic strategies are still limited. In vitro models of NAFLD are critical to understanding the pathogenesis and searching for effective therapies; thus, we evaluated the effects of free fatty acids (FFAs) on NAFLD hallmarks and their association with the modulation of Annexin A2 (ANXA2) and Keratin 17 (KRT17) in HepG2 cells. Our results show that oleic and palmitic acids can differentially induce intracellular lipid accumulation, cell death, and promote oxidative stress by increasing lipid peroxidation, protein carbonylation, and antioxidant defense depletion. Moreover, a markedly increased expression of inflammatory cytokines demonstrated the activation of inflammation pathways associated with lipotoxicity and oxidative stress. ANXA2 overexpression and KRT17 nuclear translocation were also observed, supporting the role of both molecules in the progression of liver disease. Taken together, these data provide insights into the interplay between ANXA2 and KRT17 in NAFLD, paving the way for understanding molecular mechanisms involved with the disease and developing new therapeutic strategies.

## 1. Introduction

Non-alcoholic fatty liver disease (NAFLD) is the most common chronic liver disease worldwide, affecting approximately 30% of the general population, with an increasing number of cases [1]. It encompasses a broad spectrum ranging from steatosis to non-alcoholic steatohepatitis (NASH), which correlates with a high risk of developing cirrhosis and hepatocellular carcinoma. NAFLD is strictly associated with obesity, insulin resistance, and dyslipidemia and has been considered the hepatic manifestation of the metabolic syndrome [2,3]. Although the exact molecular mechanisms that drive the pathogenesis of the disease are still not completely understood, several lines of evidence suggest the occurrence of assorted molecular events, or multiple hits, acting synergistically in the establishment and progression of NAFLD [4].

The accumulation of free fatty acids (FFAs) within hepatocytes promotes steatosis and lipotoxicity, which induce oxidative stress, endoplasmic reticulum stress, and mitochondrial dysfunction followed by reactive oxygen species (ROS) production. ROS can initiate lipid peroxidation by targeting polyunsaturated fatty acids, resulting in the formation of highly reactive aldehyde products such as 4-hydroxy-2-nonenal (4-HNE) and malondialdehyde (MDA) [5,6,7]. Thus, redox imbalance activates proinflammatory and profibrotic pathways, promoting the progression of the disease toward NASH [8,9].

Lipotoxicity caused by excessive ROS production can also lead to DNA damage. It has been demonstrated that it alters the expression of inflammatory cytokines such as interleukins IL1β, IFN-γ, IL17, IL22, and Transforming Growth Factor-β (TGFβ) [9]. This inflammatory axis upregulates Keratin 17 (KRT17), a type I intermediate filament with structural support and cell protection functions [10]. KRT17 overexpression is strongly associated with fibrotic activity and tumorigenesis [11]. Interestingly, KRT17 interacts with Annexin A2 (ANXA2), suggesting crosstalk between the two proteins regulating key events such as cell proliferation, membrane domain organization, apoptosis, angiogenesis, and inflammation [10,12]. Moreover, recent data suggest that both KRT17 and ANXA2 could be potential biomarkers for the diagnosis of liver diseases [13,14,15].

Despite lifestyle changes, the treatment of NAFLD is still limited due to the lack of effective pharmacological interventions and the absence of practical, non-invasive diagnostic approaches [8,16]. Thus, in vitro models of NAFLD represent helpful tools for evaluating the underlying mechanisms and assessing potential therapeutic compounds. HepG2 cells have been the most common immortalized cell line used for 2D in vitro models of NAFLD [17]. They can be maintained in culture for long periods in a stable manner, preserving the expression of hepatic markers and assay reproducibility [18,19]. The most well-established in vitro models of NAFLD are based on exposure to a mixture of fatty acids [20]. Although previous studies have consistently shown that the concentrations and chemical structure of FFAs play a crucial role in the development and progression of NAFLD [21], there is little consensus on experimental standardization, and studies simultaneously comparing the impact of FFAs on diverse hallmarks are lacking. Therefore, we exposed HepG2 cells to different concentrations of oleic and palmitic acids, the major FFAs in Western diets, to induce a fat overaccumulation profile in which oxidative stress and inflammation status were evaluated. Finally, we demonstrated that the transcriptional and translational levels of ANXA2 and KRT17 are also modulated, suggesting an interplay between both the proteins in NAFLD and opening a new path in understanding the molecular mechanisms underlying the pathogenesis of the disease.

## 2. Results

### 2.1. Oleic and Palmitic Acids Differentially Induce Lipid Accumulation in HepG2 Cells

**Figure 1:**
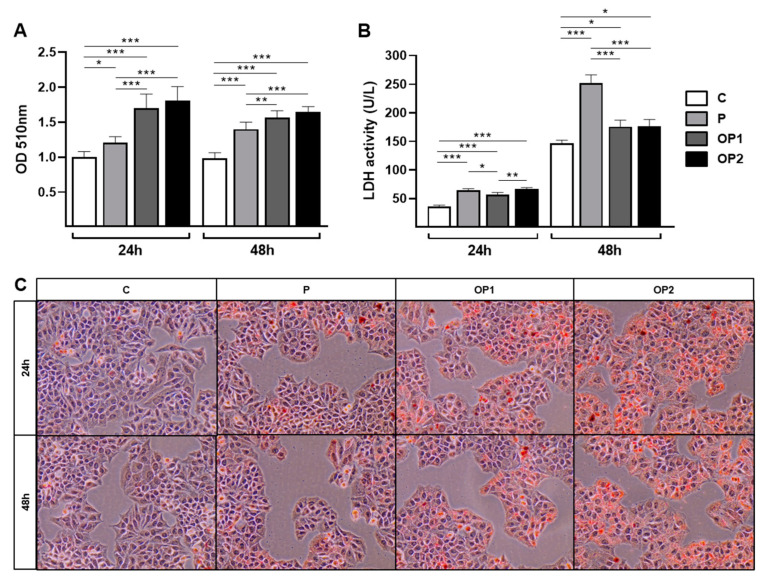
Effect of FFA-induced steatosis on intracellular lipid content and cytotoxicity in HepG2 cells. (**A**) Optical density quantification of ORO-based colorimetric assay at 510 nm after ORO staining. (**B)** Lactate dehydrogenase (LDH) activity in the supernatant of HepG2 cells exposed to lipid overload. (**C**) Representative photomicrographs of ORO and Hematoxylin staining in HepG2 cells exposed to lipid overload at 200× magnification. Data are expressed as mean ± SD (*n* = 3). * *p* < 0.05, ** *p* < 0.01, *** *p* < 0.001.

To validate the in vitro model of NAFLD, we first analyzed the development of lipid accumulation within HepG2 cells after 24 and 48 h of exposure to oleic and palmitic acids in different concentrations indicated by Oil Red O (ORO) staining (Figure 1). In the absence of FFAs, ORO staining revealed almost no intracellular lipid droplets. On the other hand, after treatment with all concentrations of FFAs, lipids accumulated within the cytoplasm of the cells, particularly and more significantly in those exposed to oleic and palmitic acids combined at 1 and 2 mM (OP1 and OP2). Lower fat levels were observed in cells exposed to palmitic acid alone at 0.7 mM (P), highlighting the more significant steatogenic effect of oleic acid. The optical density analysis showed a dose-dependent increase in fat accumulation.

To further assess the effects of FFA-induced steatosis on HepG2 cells, the cytotoxicity was determined by the activity of LDH released in the culture media (Figure 1B). A significant increase in the activity of LDH was observed in all groups exposed to FFAs after 24 h compared to the control, with P and OP2 showing slightly higher cytotoxicity when compared to OP1 as well. These results were similar to 48 h of FFA exposure, which led to increased levels of cytotoxicity when compared to 24 h. It was also observed that palmitic acid was associated with significantly higher LDH activity, indicating a palmitic acid-time-dependent manner cytotoxicity.

In summary, these results indicate that lipid exposure is efficient in inducing hepatic steatosis in cultured cells, where a wide range of experimental outcomes could be observed for the different combinations and concentrations of both FFAs.

### 2.2. Oleic and Palmitic Acids Impair Cellular Antioxidant Defense and Induce Oxidative Stress in HepG2 Cells

**Figure 2:**
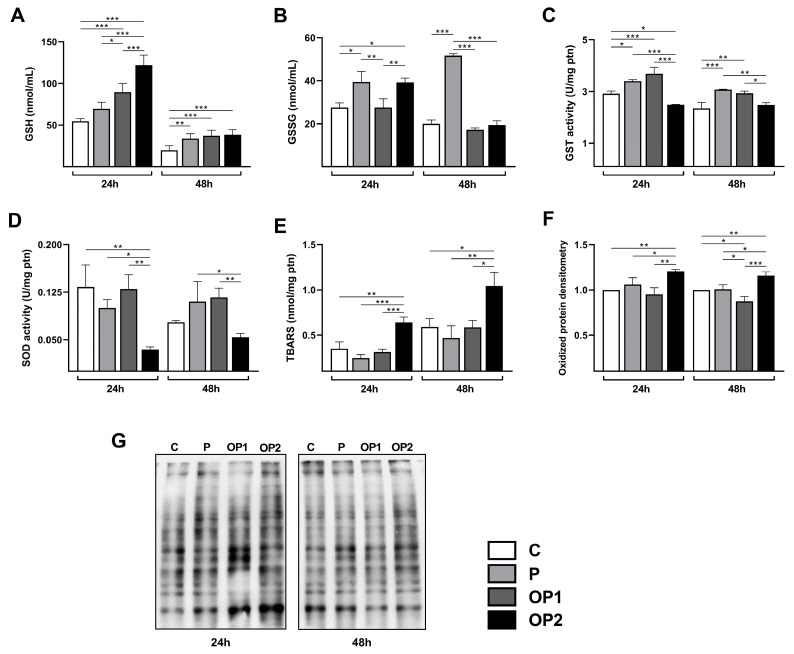
Effect of FFA-induced steatosis on antioxidant system and oxidative stress markers in HepG2 cells. Content of (**A**) GSH, reduced, and (**B**) GSSG, oxidized Glutathione. Activity of (**C**) GST, Glutathione S-transferase, and (**D**) SOD, Superoxide dismutase. Intracellular levels of (**E**) TBARS, Thiobarbituric acid reactive substances, and (**F**,**G**) total oxidized protein. Data are expressed as mean ± SD (*n* = 3). * *p* < 0.05, ** *p* < 0.01, *** *p* < 0.001.

Under physiological conditions, hepatic lipid overload induces the overproduction of oxidants by changing the redox homeostasis with several modifications to cellular macromolecules and dysregulated ROS signaling. Therefore, we next investigated whether induced steatosis may affect key components of the antioxidant system (Figure 2).

In the present study, lipid overload led to a significant increase in reduced glutathione (GSH) concentration in a dose-dependent manner after 24 h. Higher concentrations were additionally found after 48 h compared to the control group, but no difference was observed between the treated groups. When assessed, oxidized glutathione (GSSG) levels were significantly elevated in P and OP2 at 24 h. On the other hand, after 48 h, only palmitic acid stimulation significantly increased GSSG concentration compared to the control.

We next assayed the activity of Glutathione-S-Transferase (GST), a family of enzymes involved in the detoxification of endogenous and exogenous compounds, by catalyzing its conjugation with GSH. Therefore, a significant reduction in GST activity was observed in OP2, although P and OP1 led to its increase at 24 h. Despite no difference between OP2 and the control group, a higher activity was also observed for P and OP1 at 48 h.

To evaluate the effect of FFA-induced steatosis on oxidative cell damage, we measured the levels of lipid peroxidation and oxidized protein by carbonylation, which were assayed using TBARS and Oxyblot methods, respectively. The results show that lipid overload induced by OP2 induced a consistent increase in TBARS levels and carbonylated proteins after 24 and 48 h periods. In the other treated groups, the TBARS and Oxyblot values remained similar to those observed in the control group.

### 2.3. Oleic and Palmitic Acids Upregulate Transcriptional Expression of Inflammatory Cytokines

**Figure 3:**
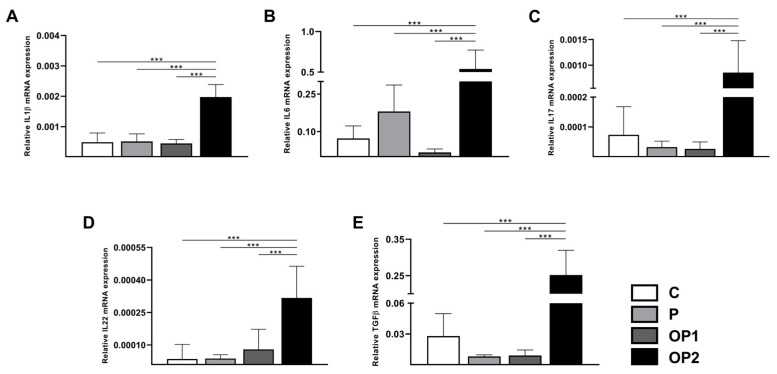
Effect of FFA-induced steatosis on mRNA expression of cytokines in HepG2 cells. Relative mRNA expression of (**A**) *IL1β*, Interleukin 1-beta; (**B**) *IL6*, Interleukin 6; (**C**) *IL17*, Interleukin 17; (**D**) *IL22*, Interleukin 22; and (**E**) *TGFβ*, Transforming growth factor beta. Data are expressed as mean ± SD (*n* = 3). *** *p* < 0.001.

Clinical approaches report that more severe stages of NAFLD are associated with the activation of inflammatory processes. Therefore, we evaluated the effects of FFA-induced steatosis on the transcriptional expression of inflammation mediators (Figure 3). It is important to mention that the following data refers to tests performed in cells after 24 h of FFA exposure because, in this period, the results were found to be more promising. A more prolonged incubation time (48 h), while not providing a significant advantage in terms of intracellular lipid accumulation or oxidative stress, was associated with a significant decrease in cell viability.

Although no significant difference was observed in P and OP1, the mRNA expression levels of *IL1β*, *IL6*, *IL17*, *IL22*, and *TGFβ* were significantly higher in OP2.

In summary, our results suggest that increasing FFA concentrations activates the inflammatory process, potentially mediated by a dysregulation of the cellular redox state. Remarkably, coincubation with oleic and palmitic acids at a 2 mM concentration displayed a more pronounced inflammatory response.

### 2.4. Oleic and Palmitic Acid Modulate KRT17 and ANXA2 Expression

**Figure 4:**
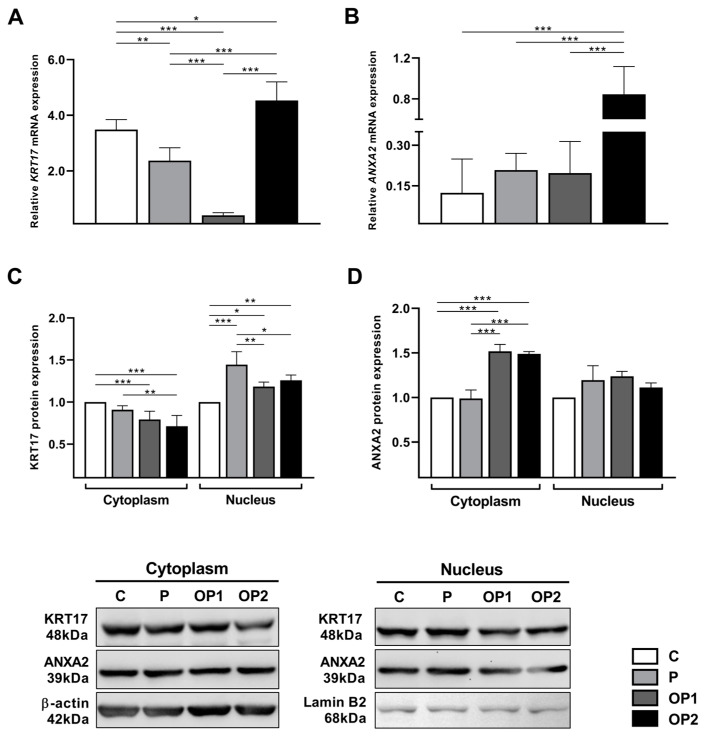
Effect of FFA-induced steatosis on transcriptional and translational expression of KRT17 and ANXA2 in HepG2 cells. Relative mRNA expression of (**A**) KRT17, Keratin 17, and (**B**) ANXA2, Annexin A2. Protein expression of (**C**) KRT17 and (**D**) ANXA2. Data are expressed as mean ± SD (*n* = 3). * *p* < 0.05, ** *p* < 0.01, *** *p* < 0.001.

To further investigate the effect of FFA-induced steatosis on KRT17 and ANXA2 expression, we assessed both the transcriptional and protein levels (Figure 4). While OP2 showed augmented *KRT17* mRNA expression compared to the control, P and OP1 *KRT17* transcripts were significantly decreased. When we assessed the protein expression and cell location of KRT17, lower cytoplasmic levels were observed for OP1 and OP2, whereas all treated groups induced a consistent increase in KRT17 nuclear expression as compared to the control, suggesting its translocation into the nucleus.

We then investigated whether FFA-induced steatosis would play a role in ANXA2 expression. Only OP2-treated cells showed an upregulation in *ANXA2* mRNA, while no difference was found in P and OP1. Although there was no significant difference in the nuclear expression of ANXA2 for any treated group, its cytoplasmic expression significantly increased in OP1 and OP2.

Overall, a higher availability of FFAs is associated with oxidative damage and the upregulation of inflammatory mediators, which may upregulate the expression of ANXA2 and the nuclear translocation of KRT17 in HepG2 cells.

## 3. Discussion

NAFLD has emerged as a global health concern associated with various comorbidities, hence referred to as a multisystemic disease. We investigated the in vitro effect of oleic and palmitic acids in the major hallmarks of NAFLD to perform a comprehensive characterization of the role of FFAs on lipid accumulation, oxidative stress, inflammatory mediators, and KRT17 and ANXA2 expression under the same experimental conditions.

Lipidomic profiling studies have shown that plasma FFA concentrations are around 1 mM in patients with NAFLD [22,23]. In an interesting examination of the serum FFA composition in the postprandial state, scientists reported increased levels in NAFLD patients, reaching 1.4 mM after fat uptake mainly due to oleic and palmitic acids, which are the most abundant circulating FFAs [24]. Therefore, the FFA concentration employed in our study mimics human dietary conditions. Furthermore, most in vitro studies use acute FFA intake concentrations ranging from 0.3 up to 3 mM in various combinations, showing that higher concentrations can lead to increased lipid accumulation [25,26] and making it more evident to observe other hallmarks of NAFLD.

Our findings demonstrate that different FFA concentrations induce a dose-dependent increase in lipid droplet content. Coincubation with oleic and palmitic acids has also proven more efficient than isolated FFA overload, which is consistent with previous studies [21,27]. These FFAs act as precursors in triglyceride synthesis, which are stored in the cytosol as micro- or macrovesicular steatosis. Additionally, triglycerides are the main type of fat in lipid droplets, and their formation is considered a protective mechanism against lipotoxicity in hepatocytes [25]. The higher increase in fat accumulation found in cells treated with oleic and palmitic acids combined is attributable to the more effective steatogenic property of oleic acid, as unsaturated fatty acids are more readily esterified into triglycerides.

When the lipid storage capacity of hepatocytes is exceeded, FFAs may enter deleterious pathways, causing cell damage and death. Our study revealed that the exposure of HepG2 cells to all combinations of oleic and palmitic acids resulted in increased lactate dehydrogenase (LDH) leakage, indicating the disruption of cytoplasmic membrane integrity and cell death. Notably, palmitic acid alone exhibited significantly higher cytotoxicity, corroborating previous reports of its cytotoxic effects across various concentrations and cell lines [21,25,28,29]. Additionally, all treatments showed significantly higher rates at 48 h, suggesting that LDH release depends on the duration of FFA exposure. The increased availability of FFAs also promotes lipid oxidation via mitochondrial or alternative pathways [30], which drives a significant increase in ROS production and oxidative stress [31]. Clinical studies of NAFLD often report decreased concentrations or activities of enzymatic and non-enzymatic antioxidants, although discrepancies exist based on the type of sample and disease stage. In our study, the highest concentration of combined FFAs induced significant alterations in the antioxidant system. The GSH and GSSG levels were found to be increased, indicating an adaptive response mechanism to oxidative stress. In contrast, SOD and GST activities were notably reduced, evidencing a depletion in the cellular antioxidant system.

Oxidative stress biomarkers are generally elevated in patients and experimental models of NAFLD. Lipid overload often triggers lipid peroxidation in steatotic cells and oxidative damage to several cellular biomolecules [32,33,34,35]. Our results demonstrate that higher concentrations of FFAs lead to an increase in lipid peroxidation levels. Consistent with these data, protein oxidation was also elevated under the same conditions. Multiple studies have documented the deleterious effect of lipid peroxides on molecular targets, causing damage in both intra- and extracellular spaces. As one of the main targets, the formation of carbonyl groups in protein side chains by its reaction with pro-oxidants and lipid peroxidation intermediates, especially in proline, arginine, lysine, and threonine residues, is a common biochemical marker of oxidative stress [36,37,38].

Current evidence indicates that lipotoxicity plays a pivotal role in activating multiple inflammatory pathways. We therefore reported the overexpression of several cytokines in cells treated with a concentration of 2 mM of FFA mixture. As a critical hallmark of NAFLD, immune cells typically increase at the transition from steatosis to NASH, leading to fibrosis through the release of cytokines like IL-6, IL-1β, IL-17, IL-22, TGF-β, and tumor necrosis factor-α (TNF-α). The pathogenetic role of IL-17 in the progression from fatty liver to NASH via JNK activation has also been documented in other studies [39]. Conversely, emerging evidence indicates the protective role of IL-22 against NAFLD by improving antioxidant status and liver injury, primarily mediated by promoting STAT3 phosphorylation [40]. Furthermore, these cytokines and growth factors upregulate KRT17 expression [10].

In our study, the mixture of oleic and palmitic acids promoted a cytoplasmic decrease in KRT17 protein levels. At the same time, its nuclear rate was markedly overexpressed in all treated groups, evidencing the translocation of KRT17 into the nucleus. KRT17 can be found in the nucleus in response to DNA damage, modulating gene expression associated with inflammation, nuclear architecture, and proliferation [41,42]. The overexpression of KRT17 induces the proliferation, migration, and activation of hepatic stellate cells by increasing TGF-β signaling in liver fibrosis [11]. KRT17 has been associated with lipid accumulation and fibrosis progression in HepG2 cells treated with Trimethylamine N-oxide (TMAO), which is a gut microbiota metabolite [43]. Previous studies have also documented the correlation between KRT17 and redox signaling. Nrf2, a transcription factor known to modulate cellular defense against oxidative stress, has been identified as a positive regulator of KRT17 expression [44].

The interplay between KRT17 and ANXA2 has already been described, although the precise nature of the interaction remains elusive. In our study, FFA-induced steatosis upregulated the expression of ANXA2 mRNA and cytoplasmic protein levels in HepG2 cells treated with oleic and palmitic acids; however, it did not affect the nuclear levels of ANXA2. Clinical studies have reported the critical role of ANXA2 as an intermediary protein connecting abnormal inflammation with liver injury and fibrosis in NAFLD progression. Indeed, ANXA2 is notably upregulated in serum and liver samples from patients and mice with NAFLD, particularly in NASH livers [14,15]. In addition, ANXA2 depletion can attenuate diet-induced steatosis and liver injury in mice [15]. It is worth mentioning that ANXA2 also plays an essential role in cellular redox regulation; it is often upregulated in response to oxidative stress and hypoxia, and its depletion is associated with higher levels of oxidized proteins [45]. ANXA2 exists in multiple cellular locations. It is mainly soluble in the cytoplasm, linked to the actin cytoskeleton, or associated with the plasma membrane, but it can also be located within the nucleus [46]. The nuclear levels of ANXA2 vary according to the cell cycle and seem to be associated with phosphorylation at Ser25 residue. Within the nucleus, ANXA2 acts as a primer recognition protein complex component in DNA replication and protects DNA from damage [47]. It was previously reported that KRT17 influences ANXA2 phosphorylation by acting as a scaffold and affecting its distribution and/or allocation. Our findings suggest that the decreased cytoplasmic levels of KRT17 could affect the phosphorylation of ANXA2 and, therefore, hamper its translocation to the nucleus.

In summary, our data show that lipid accumulation induced by oleic and palmitic acids mixture is consistently associated with oxidative stress, inflammation, and the overexpression of ANXA2 and KRT17 in HepG2 cells. Interestingly, although both tested FFA mixtures led to similar fat accumulation levels, their effects on key biomarkers of NAFLD features were notably different. While most in vitro models of NAFLD are suitable for assessing the impact of lipid overload on singular features of the disease, important events are often excluded. Therefore, studies that embrace more features are essential to better replicate in vivo conditions, providing a valuable and more sustainable tool for clinical and pharmaceutical investigation. Our findings also reveal, for the first time, the nuclear translocation of KRT17 associated with NAFLD. Nonetheless, further investigations are required to fully elucidate the underlying mechanisms. Our study supports KRT17 and ANXA2 as potential biomarkers and therapeutic targets for NAFLD.

## 4. Materials and Methods

### 4.1. Reagents

Dulbecco’s Modified Eagle Medium low glucose (DMEM), fetal bovine serum (FBS), and penicillin–streptomycin were purchased from Thermo Scientific (Waltham, MA, USA). Trypsin, fatty acid-free bovine serum albumin, oleic acid, palmitic acid, and Oil Red O were from Sigma-Aldrich (St. Louis, MO, USA).

### 4.2. Cell Culture

HepG2 cell line was obtained from the Cell Bank of Rio de Janeiro. Cells were cultured in DMEM supplemented with 10% FBS and 1% penicillin–streptomycin under a 5% CO_2_ humid atmosphere at 37 °C. For subculturing purposes, HepG2 cells were detached with 0.25% trypsin-EDTA at 37 °C at a frequency of 2 to 3 days. Cultures were used at 80% confluency.

### 4.3. FFA-Induced Steatosis and Cytotoxicity

To induce lipid accumulation, HepG2 cells were seeded in appropriate dishes with supplemented media for adhesion. After starvation with unsupplemented media for 24 h, cells were exposed to different ratios of long-chain FFAs conjugated to 4.5% fatty acid-free bovine serum albumin (palmitic at 0.7 mM (P) or oleic and palmitic acids at final concentrations of 1 (OP1) and 2 mM (OP2) in a 2:1 ratio). For this purpose, stock solutions of the oleic and palmitic acids were prepared at concentrations of 150 mM diluted in ethanol and stored at −20 °C until usage, when they were diluted to working solutions of 3 mM in DMEM conjugated to 4.5% BSA. Ethanol was used as a vehicle solution in the control group (C). After 24 and 48 h incubation, steatotic or control cells were collected and used in the following assays.

To assess intracellular lipid content, cells were fixed in 4% paraformaldehyde for 15 min and then stained with 60% Oil Red O (ORO) for 50 min at room temperature. For qualitative analysis, the stained cells were washed with distilled water and incubated with 60% isopropanol to dissolve the ORO reagent, and this solution was measured at 510 nm. For microscopic analysis, after staining with ORO, the cells were also stained with Hematoxylin for better visualization of the nuclei. Photomicrographs were then captured under a light microscope at 200× magnification.

Cytotoxicity was determined by the activity of lactate dehydrogenase (LDH), which was assessed using a commercial kit from Labtest Diagnostica (Lagoa Santa, Brazil). For that purpose, HepG2 cells were cultured in phenol red-free media, as well as the dilution of oleic and palmitic acids. After 24 and 48 h of lipid exposure, the supernatant was aspired and used in the assay.

### 4.4. Endogenous Antioxidant System

To investigate the effect of FFA-induced steatosis on the endogenous antioxidant system and oxidative stress, we assessed the content of reduced (GSH) and oxidized glutathione (GSSG), as well as the activities of enzymes glutathione S-transferase (GST) and superoxide dismutase (SOD). GSH and GSSG content were determined using a kinetic assay [48]. Briefly, cells were solubilized in extraction buffer (0.1% Triton-X and 0.6% sulfosalicylic acid in 0.1 M potassium phosphate buffer (KPE) with 5 mM EDTA disodium salt). After freeze/thaw cycles, the cells were centrifuged at 3000× *g* for 4 min at 4 °C, and the supernatant was used in the assay. The samples were added to a 96-well microplate, followed by a working mixture (KPE, DTNB, and glutathione reductase) and NADPH for an absorbance reading at 405 nm every 30 s for 2 min. For GSSG, 2 μL of 2-vinylpyridine (1:10) was added to 100 μL of the cell homogenate, followed by 4 μL of triethanolamine (1:6) after 1 h of incubation; then, the derivative samples were assayed as described above. Reduced and oxidized glutathione salts were used to build standard curves.

The enzymatic activity of GST was assessed using the method based on the conjugation of CDNB (Sigma-Aldrich) with reduced glutathione (Sigma-Aldrich), as described by Habig et al. [49]. Briefly, treated cells were collected in PBS using a cell scraper and lysed through 3 freeze/thaw cycles, and the supernatant was collected after centrifugation for 15 min at 10,000× *g* at 4 °C. The sample was added to the reaction mix (100 mM phosphate buffer, pH 6.5, with 0.1% Triton X-100 and 100 mM reduced glutathione in deionized water). To start the reaction, 10 uL of 100 mM CDNB in 95% ethanol was added. Absorbances were determined at 340 nm using a spectrophotometer every minute for 5 min. All analyses were performed in triplicate. The results were normalized by protein concentration, determined using the Bradford method [50], and expressed as units per mg of protein (U/mg pt).

SOD activity was determined by monitoring the autoxidation of pyrogallol along with the SOD-dependent reduction of MTT [51]. Briefly, samples were added to a microplate with phosphate buffer 50 mM pH 7.0, 1.25 mM MTT, and 100 μM of pyrogallol. DMSO was added to stop the reaction, and the absorbance was assessed using a microplate reader. One unit of SOD activity is defined by the amount of enzyme needed to reduce the absorbance of formazan salt by 50%, and the results are expressed as units per mg of protein (U/mg ptn).

### 4.5. Oxidative Stress

The effect of FFA-induced steatosis on cellular oxidative stress was determined by the levels of lipid peroxidation and protein carbonylation. The levels of thiobarbituric acid reactive substances (TBARSs) were estimated using the method of Buege and Aust [52] as an index of lipid peroxidation. For that purpose, treated cells were harvested, lysed, and mixed with TCA, TBA, and BHT; heated for 15 min at 95 °C; and then placed in an ice bath. Precipitated material was removed using centrifugation, and the absorbance of the clear supernatant was read at 535 nm.

To assess protein carbonylation by Oxyblot, protein samples were derivatized in a solution containing 10 mM 2,4-dinitrophenylhydrazine-10% trifluoracetic acid, as described by Levine et al. [53]. The reaction was stopped immediately after 20 min of incubation through neutralization with 2 M Tris base and 30% glycerol, and it was then separated by 10% SDS-PAGE with 5 μg of protein load per track. For immunodetection using western blotting, the gel was incubated with a primary rabbit anti-2,4-dinitrophenol antibody (1:2000; Sigma-Aldrich D9656) and a secondary anti-rabbit IgG-HRP antibody (1:5000; Santa Cruz Biotechnology, Dallas, TX, USA, sc2370). After revelation with ECL, carbonylated proteins were measured using densitometry, as described below.

### 4.6. qPCR Analysis

Total RNA was extracted using TRIzol reagent (Invitrogen, Waltham, MA, USA) according to the manufacturer’s recommendations. The RNA concentration and quality were analyzed in 1.5 % agarose gel stained with GelRed 1× (Uniscience, Sao Paulo, Brazil) and spectrophotometrically using absorbance readings (NanoDrop Lite—Thermo Scientific). cDNA was then synthesized from 1 μg of total RNA using Moloney Murine Leukemia Virus Reverse Transcriptase (Sigma Aldrich), according to the manufacturer’s instructions, and stored at −20 °C.

The real-time PCR (qPCR) was conducted in an ABI PRISM 7300 Sequence Detection System (Applied Biosystems, Carlsbad, CA, USA) using Power SYBR Green PCR Master Mix (Applied Biosystems). Cycling conditions were performed at 95 °C for 10 min, followed by 40 cycles of 95 °C for 15 s and 60 °C for 60 s, except for *IL17* and *IL22*, wherein their annealing temperatures were 62 °C to increase specificity. Expression levels were normalized to the beta-2-microglobulin (β2M) transcripts. After optimization of the standard comparative curve, the gene expression was calculated using the comparative Cq method. Oligonucleotide sequences used for qPCR are presented in Table 1.

### 4.7. Western Blot

Protein extracts were obtained using RIPA lysis buffer (50 mM TRIS-HCl pH 8.0, 150 mM NaCl, 1% Triton X-100, and 0.5% sodium deoxycholate) supplemented with Protease and Phosphatase Inhibitor Cocktail (Sigma Aldrich) or NE-PER Nuclear and Cytoplasmic Extraction Reagent Kit (Thermo Scientific) according to the manufacturer’s protocol. Total protein concentration was measured by the Bradford assay. Proteins were loaded onto 12% sodium dodecyl sulfate–polyacrylamide gels for electrophoresis and then transferred to nitrocellulose membranes. The membranes were blocked with 5% non-fat milk at room temperature for 1 h on a shaker and incubated with primary antibodies anti-KRT17 (1:1000; Sigma Aldrich SAB4501662) and anti-ANXA2 (1:2000; Life Technologies, Carlsbad, CA, USA, #034400) overnight at 4 °C. Anti-β-actin (1:3000; Sigma Aldrich A1978) and anti-lamin B2 (1:8000; Proteintech, Rosemont, IL, USA, #10895) were used as labeling controls for cytoplasmic and nuclear extracts, respectively, at room temperature for 1 h. After washing, HRP conjugated secondary antibodies (1:5000 bovine anti-rabbit IgG-HRP—Santa Cruz Biotechnology sc2370—or goat anti-mouse IgG-HRP—Sigma-Aldrich A4416) were added for 1 h and washed for the visualization of protein bands, which was accomplished through enhanced chemiluminescence followed by densitometry analysis in ImageJ software v. 1.53k (Bedestha, MD, USA).

### 4.8. Statistical Analysis

Data normality was tested using the Kolmogorov–Smirnov test. Parametric data were analyzed using one-way analysis of variance (ANOVA) followed by the Tukey test and expressed as the mean ± standard deviation (SD). Differences were considered significant for *p* < 0.05. All analyses were conducted using GraphPad Prism version 8.0.1 software for Windows (San Diego, CA, USA).

## Figures and Tables

**Table 1:** Oligonucleotide sequences used for qPCR assays.

Primer	Sequence 5′-3′	Amplicon (bp)
*IL1β* forward	CAGGATATGGAGCAACAAGTGG	136
*IL1β* reverse	GGGCTTATCATCTTTCAACACGC	
*IL17* forward	CAATCCCACGAAATCCAGGATG	156
*IL17* reverse	GGTGGAGATTCCAAGGTGAGG	
*IL22* forward	CTGATAACAACACAGACGTTCG	170
*IL22* reverse	CCACCTCCTGCATATAAGGC	
*TGFβ* forward	GTACCTGAACCCGTGTTGCTC	107
*TGFβ* reverse	CAGGAATTGTTGCTGTATTTCTGG	
*IL6* forward	GATTCCAAAGATGTAGCCGCC	242
*IL6* reverse	ATTTTCACCAGGCAAGTCTCCTC	
*KRT17* forward	CTGATGACTTCCGCACCAAGTT	234
*KRT17* reverse	CAGCGTCCATCTCCACATTG	
*ANXA2* forward	TGACCAAGATGCTCGGGATC	113
*ANXA2* reverse	TTTCTGGAGGTGGGGCA	
*BM2* forward	AGCAGAGAATGGAAAGTCAAA	94
*BM2* reverse	TGTTGATGTTGGATAAGAGAA	

## Data Availability

The raw data supporting the conclusions of this article will be made available by the authors upon request.
